# Single-Arm, Prospective, Interventional Study of *Helicobacter pylori* Eradication Rescue Therapy with Rifabutin, Metronidazole, and Vonoprazan

**DOI:** 10.3390/jcm13133774

**Published:** 2024-06-27

**Authors:** Soichiro Sue, Ryosuke Ikeda, Aya Ikeda, Hiroki Sato, Hiroaki Kaneko, Kuniyasu Irie, Shin Maeda

**Affiliations:** Department of Gastroenterology, Yokohama City University Graduate School of Medicine, Yokohama 236-0004, Japan

**Keywords:** 10-day triple therapy, *Helicobacter pylori*, metronidazole, rifabutin, vonoprazan

## Abstract

**Background and Objective:** Rescue *Helicobacter pylori* eradication can be challenging. Rifabutin (RBT) demonstrates high activity against *Helicobacter pylori* and is incorporated into various rescue eradication regimens. This exploratory study was performed to evaluate the efficacy and safety of a rescue regimen comprising RBT, metronidazole (MNZ), and vonoprazan (VPZ). **Methods:** This prospective, single-center, single-arm, interventional study was performed in Japan. Eligible patients were those who underwent failed primary eradication treatment (7-day treatment with three drugs: VPZ or a proton pump inhibitor [PPI], amoxicillin [AMPC], and clarithromycin) and secondary eradication treatment (7-day treatment with three drugs: VPZ or a PPI, AMPC, and MNZ) and those who were unable to receive first- and second-line therapy because of penicillin allergy. Twenty *Helicobacter pylori*-positive patients were treated with RBT (150 mg twice daily), MNZ (250 mg twice daily), and VPZ (20 mg twice daily) for 10 days (RBT-MNZ-VPZ therapy). Eradication success was evaluated using the urea breath test. Drug susceptibility test results were available in 16 patients. This study is registered in the Japan Registry of Clinical Trials (jRCT031220504). **Results:** The intention-to-treat (ITT) and per-protocol (PP) eradication rates of RBT-MNZ-VPZ therapy were 70% (90% confidence interval [CI]: 49.2%–86.0%) and 72.2% (95% CI: 50.2%–88.4%), respectively. In the MNZ-susceptible subgroup, the ITT (*n* = 8) and PP (*n* = 7) eradication rates were 100% (90% CI: 68.8%–100%) and 100% (90% CI: 65.2%–100%). In the MNZ-resistant subgroup, the ITT (*n* = 8) and PP (*n* = 7) eradication rates were both 62.5% (90% CI: 28.9%–88.9%). All infections were RBT-susceptible. **Conclusions:** These findings suggest that RBT-MNZ-VPZ therapy may be a promising rescue regimen, especially in MNZ- and RBT-susceptible infections or patients with penicillin allergy.

## 1. Introduction

*Helicobacter pylori* infection contributes to gastric carcinogenesis by several mechanisms [1,2], and its eradication is necessary to reduce the incidence of gastric cancer [3,4]. Therefore, effective eradication regimens are important to ensure successful treatment.

The national health insurance system in Japan has approved a first-line eradication regimen for *H. pylori*-associated gastritis: 7 days of treatment with vonoprazan (VPZ), which is a potassium-competitive acid blocker, or proton pump inhibitor (PPI)-based triple therapy with amoxicillin (AMPC) and clarithromycin (CAM). The approved second-line eradication regimen is VPZ- or PPI-based triple therapy with AMPC and metronidazole (MNZ). VPZ-based regimens reportedly have higher eradication rates than PPI-based regimens, not only in first- and second-line therapies but also in patients for whom first- and second-line therapies have failed and in patients with penicillin allergy [5]. Therefore, in Japan, VPZ is usually used as an antacid for *H. pylori* eradication.

We previously reported the results of eradication therapy for patients who had undergone failed second-line therapy and for patients with penicillin allergy (so-called rescue therapy). Our data revealed an eradication rate of 75.8% (95% CI: 57.7%–88.9%) with VPZ, AMPC, and sitafloxacin (STFX) triple therapy [6]. Achieving an eradication rate of 90%–95% in third-line therapy will require another option beyond STFX-based therapy, especially for STFX-resistant infections. Additionally, among patients with penicillin allergy, a VPZ-based regimen with CAM and MNZ yielded high eradication rates as a first-line therapy [7,8], but to date, no second-line regimen has been established.

Rifabutin (RBT) is a rifamycin S derivative with potent activity against *H. pylori*, making it a key component in various rescue eradication regimens for this infection. Unlike other antibiotics, RBT does not share cross-resistance with CAM, MNZ, or newer quinolones such as levofloxacin. Additionally, the resistance rate of *H. pylori* to RBT remains low. Therefore, RBT is a strong candidate for use as a key drug in rescue eradication regimens. Gisbert [9] reviewed the use of RBT as an antibiotic for *H. pylori* eradication, including its mechanism of action, pharmacokinetics, *H. pylori* resistance rates, efficacy of various RBT-based regimens, optimization strategies for these regimens, and safety profile. Gisbert found that the RBT resistance rate of *H. pylori* is 0.13% (*n* = 9721), and the mean *H. pylori* eradication rate (intention-to-treat [ITT] analysis) is 73% (*n* = 3052). Most studies administer RBT at 300 mg/day, which seems to be more effective than 150 mg/day. A treatment duration of 10 to 12 days is generally recommended, and severe adverse events are exceptional and reversible. The main RBT-containing regimens reported to date are triple therapy regimens containing RBT, AMPC, and PPIs [9]. In Japan, Hirata et al. reported an eradication rate of 100% (95% CI: 83%–100%) and high safety of 10 days of triple therapy with RBT (150 mg twice daily), AMPC (750 mg twice daily), and VPZ (20 mg twice daily) [10]. These findings suggest that a VPZ-based RBT-containing regimen is more effective than a PPI-based RBT-containing regimen.

In the context of *H. pylori* rescue treatment, regimens containing VPZ and AMPC failed more than twice after the first- and second-line treatments covered by national insurance in Japan. Rescue regimens must also be developed for patients with penicillin allergy. Our current rescue regimen for penicillin-allergic patients includes the triple therapy of VPZ, MNZ, and STFX, and has yielded an 88.2% eradication rate (95% CI: 63.6%–98.5%) demonstrating the efficacy of MNZ-containing regimens even after first-line treatment failure [11]. Therefore, we conducted this study to assess a new RBT-based regimen with VPZ and MNZ as a rescue treatment for patients who previously underwent national insurance-covered eradication treatments or were unable to undergo these treatments.

## 2. Materials and Methods

### 2.1. Study Design

This single-center, open-label, single-arm intervention study was designed to assess a 10-day course of triple therapy with RBT, MNZ, and VPZ (RBT-MNZ-VPZ therapy). The research was carried out in compliance with Japan’s Clinical Trial Act and the Declaration of Helsinki and was conducted at Yokohama City University Hospital. The study protocol was reviewed and approved by the Yokohama City University Certified Institutional Review Board (CRB No. CRB3180007) on 1 December 2022 (Approval No. CRB22-004). This study was registered in the Japan Registry of Clinical Trials (jRCT) on 9 December 2022 (identifier jRCT031220504), as mandated by the government of Japan in its 2019 Clinical Trials Act. All study participants provided informed written consent prior to study enrollment.

### 2.2. Study Population

The inclusion criteria for this study were a diagnosis of *H. pylori* infection by a positive urea breath test (UBT) [12], positive *H. pylori* stool antigen test [13], positive *H. pylori* culture [14], or positive rapid urease test [15]; a history of failed first-line therapy with a national insurance-covered eradication regimen (7-day triple therapy with VPZ or PPI + AMPC + CAM) and failed second-line therapy with a national insurance-covered eradication regimen (7-day triple therapy with VPZ or PPI + AMPC + MNZ) (third-line or later); a history of penicillin allergy with the inability to eradicate *H. pylori* with AMPC-containing national insurance-covered first- and second-line eradication regimens; and provision of written informed consent.

Patients who met any of the following criteria were excluded from the study: a history of allergy to RBT and rifampicin; use of voriconazole, grazoprevir, elbasvir, ticagrelor, artemether, lumefantrine, rilpivirine, emtricitabine, tenofovir alafenamide, atazanavir sulfate, or rilpivirine hydrochloride; a history of allergy to MNZ; brain or spinal cord disease; pregnancy or lactation; diagnosis of VPZ allergy; severe liver or renal dysfunction; and ineligibility as deemed by a physician.

### 2.3. Procedure

**Figure 1:**
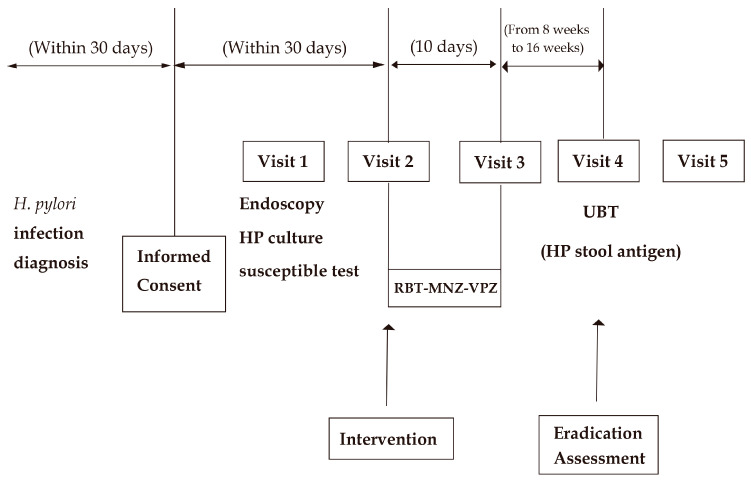
Flow diagram of study design.

As shown in the flow chart in Figure 1, this study was designed to obtain as much information regarding the antibiotic sensitivity of *H. pylori* as possible before beginning the RBT-MNZ-VPZ eradication therapy. Upper gastrointestinal endoscopy and *H. pylori* culture and susceptibility testing were performed after study registration, unless *H. pylori* culture and susceptibility testing had been conducted within 1 year before registration and no *H. pylori* eradication therapy had been performed thereafter. RBT-MNZ-VPZ eradication therapy was performed regardless of whether the *H. pylori* culture and susceptibility results were available. Each patient underwent medical examination before and after the eradication therapy. A UBT was performed 8 to 16 weeks after the eradication therapy. A medical examination was also conducted when the patient was given their UBT result. In patients with a UBT result of ≥2.5‰ to <5.0‰, an additional *H. pylori* stool antigen test was performed.

Written consent for this study was obtained for all treatments, tests, and visits after informed consent in Figure 1, including endoscopy and HP culture susceptible testing.

RBT-MNZ-VPZ: 10 days of triple therapy with rifabutin, metronidazole, and vonoprazan; Endoscopy HP culture susceptible test: upper gastrointestinal endoscopy and *Helicobacter pylori* culture and susceptibility testing; UBT: urea breath test; HP stool antigen: *Helicobacter pylori* stool antigen test.

### 2.4. Treatment

Patients received RBT 150 mg twice daily (300 mg/day), MNZ 250 mg twice daily (500 mg/day), and VPZ 20 mg twice daily (40 mg/day) for 10 days (RBT-MNZ-VPZ therapy). These dosages and the treatment duration were determined based on those found to be most useful in past studies [9,16]. RBT (rifabutin) is a semi-synthetic ansamycin antibiotic that exerts its antibacterial activity by inhibiting DNA-dependent RNA polymerase. MNZ (metronidazole) is a nitroimidazole antibiotic that exerts its antibacterial activity by disrupting DNA synthesis and electron transport in anaerobic microorganisms [17].

During treatment, all patients were prohibited from using PPIs, antibiotics other than those in the assigned regimen, and any drugs that were contraindicated with RBT, MNZ, and VPZ, as specified in the package insert. Prohibited drugs included voriconazole, grazoprevir, elbasvir, ticagrelor, artemether/lumefantrine, rilpivirine/tenofovir, alafenamide/emtricitabine, atazanavir sulfate, and rilpivirine hydrochloride.

This study was conducted in an open-label manner because the primary endpoint (the eradication rate) was based on objective data.

### 2.5. Outcome

We explored the efficacy and safety of the regimen: the primary endpoint was the eradication success rate, evaluated using the full analysis set (FAS). Eradication success was defined as a UBT result of <2.5 or a UBT result of 2.5 to <5.0 combined with a negative *H. pylori* stool antigen test. The FAS included all patients who initiated the eradication therapy, with the exception of those who did not meet the inclusion criteria; patients who were lost to follow-up or did not undergo a UBT after eradication therapy were considered treatment failures in the FAS analysis.

The secondary endpoints were eradication success rate (determined using the per-protocol set [PPS]) and safety. The PPS included patients who had no significant deviations from the research protocol in terms of dosage, administration schedule, and other criteria, and who were tested to determine eradication success.

We also preplanned subgroup analyses for patients who underwent third-line or later treatment and patients with penicillin allergy.

### 2.6. Treatment Compliance and Safety

Safety was evaluated by a physician using the Common Terminology Criteria for Adverse Events v5.0 (CTCAE). Safety was assessed using the safety analysis set, defined as patients who were enrolled in this study and received the study drugs at least once.

Safety was evaluated using an adverse effects questionnaire (AEQ) completed by patients during the therapy. To ensure comparability with our previous studies, the same AEQ was used [6,18,19]. The AEQ included questions about diarrhea, dysgeusia, nausea, anorexia, abdominal pain, heartburn, urticaria, headache, abdominal fullness, eructation, vomiting, fatigue, and other symptoms. Patients rated each symptom as none (AEQ score of 0), weak (AEQ score of 1), moderate (AEQ score of 2), or strong (AEQ score of 3). The AEQs were completed by patients and collected at the beginning of the clinical evaluation to prevent reporting bias.

Treatment adherence was assessed by counting the remaining tablets during the follow-up visit after the completion of the regimen. Poor compliance was defined as taking <80% of the trial medications.

### 2.7. Antibiotic Susceptibility Testing

Gastric biopsies were collected during endoscopy before eradication therapy. *Helicobacter pylori* culture and susceptibility testing involved an agar dilution test, as in our previous study [19]. The thresholds for antibiotic resistance of *H. pylori* are defined as 0.5 μg/mL for AMPC [20], 1.0 μg/mL for CAM [20], 8.0 μg/mL for MNZ [20], 0.12 μg/mL for STFX [21], and 0.25 μg/mL for RBT [22].

Biopsy specimen acquisition and culture sensitivity testing were included in the approved protocol for this study and were performed based on written consent to the study.

### 2.8. Sample Size Calculation

At the time of our study design, no data were available on the eradication of *H. pylori* with the RBT-MNZ-VPZ regimen. We set the threshold response rate at 75%, which is the eradication rate for the most common salvage treatments (VPZ, AMPC, and STFX) [6]. We set the expected response rate at 96% based on a similar RBT-based regimen in Japan: Hirata et al. reported a 100% response rate [10], and Inokuchi et al. reported a 91.2% response rate [23]. We set the statistical power at 80% (1 − β) and set the significance level at 5% using a two-sided test. Assuming a 10% rate of loss to follow-up, the sample size was calculated to be 20 patients.

### 2.9. Statistical Analysis

Eradication rates were calculated with the 90% confidence interval (CI). We determined whether the lower limit of the 90% CI exceeded 75%. This analysis was performed for the FAS population as the main analysis. We also performed this analysis for the PPS population, the MNZ-susceptible population, and the MNZ-resistant population as preplanned sub-analyses. Data are expressed as means ± standard deviations. All statistical analyses were performed using SPSS ver. 28 (IBM Corp., Armonk, NY, USA).

## 3. Results

### 3.1. Recruitment and Follow-Up

We registered this study in the jRCT in December 2022. Patients were enrolled beginning in January 2023. The last patient was enrolled in May 2023. The last follow-up date was September 2023.

Twenty patients with *H. pylori* infection were enrolled in the study and assigned to 10 days of RBT-MNZ-VPZ therapy. Two patients were excluded from the PP analysis: one was excluded because of a delay in the evaluation time of the UBT, and the other was excluded because of their low compliance with RBT-MNZ-VPZ therapy. The compliance rate was 100% in 19 patients and 50% in one patient.

### 3.2. Baseline Characteristics

Table 1 lists the baseline characteristics of patients. The main endoscopic finding was gastritis in all patients. Diagnosis of *H. pylori* infection was based on the UBT result (80%), *H. pylori* stool antigen test result (15%), or *H. pylori* culture result (5%). In one patient, the *H. pylori* culture and susceptibility test results for AMPC, CAM, MNZ, and STFX were obtained within 1 year before enrollment in the study. *Helicobacter pylori* culture was performed in the remaining 19 patients, and the result was positive in 16 patients. We collected information about AMPC, CAM, MNZ, and STFX resistance for these 16 patients from the *H. pylori* culture and agar plate dilution results before eradication therapy began. Resistance rates were the following: 0.0% for AMPC, 87.5% for CAM, 50.0% for MNZ, 68.8% for STFX, and 0.0% for RBT. Multi-resistance rates were 37.5% for MNZ, CAM, and STFX; 12.5% for MNZ and CAM; and 31.3% for CAM and STFX. Single resistance rates were 6.3% for CAM. All strains sensitive to MNZ, CAM, and STFX comprised 12.5%. Other multi-resistance combinations involving MNZ, CAM, and STFX were 0.0%. RBT resistance information was available for 15 patients, and *H. pylori* was sensitive to RBT in all of these patients.

SE, standard error; SD, standard deviation; UBT, ^13^C-urea breath test; CI, confidence interval; BMI, body mass index; T-Bil, total bilirubin; AST, aspartate transaminase; ALT, alanine transaminase; Cr, creatinine; FAS, full analysis set; PPS, per-protocol set; AMPC, amoxicillin; CAM, clarithromycin; MNZ, metronidazole; STFX, sitafloxacin; RBT, rifabutin; MNZ-R, metronidazole resistance; CAM-R, clarithromycin resistance; STFX-R, sitafloxacin resistance.

### 3.3. Efficacy

The ITT and per-protocol (PP) eradication rates of RBT-MNZ-VPZ therapy were 70% (90% CI: 49.2%–86.0%, *n* = 20) and 72.2% (90% CI: 50.2%–88.4%, *n* = 18), respectively. The lower limit of the 90% confidence interval did not exceed 75% in either group. Eradication success was determined using the UBT in 18 patients and both the UBT and *H. pylori* stool antigen test in two patients, as the UBT results ranged from ≥2.5 to <5.0. This definition was based on our criteria for eradication success.

The UBT was performed 11.9 ± 2.4 weeks after the RBT-MNZ-VPZ eradication therapy had been completed. In the MNZ-susceptible subgroup, the ITT and PP eradication rates were 100% (90% CI: 68.8%–100%, *n* = 8) and 100% (90% CI: 65.2%–100%, *n* = 7), respectively. In the MNZ-resistant subgroup, the ITT and PP eradication rates were both 62.5% (90% CI: 28.9%–88.9%, *n* = 8). RBT sensitivity was observed in all patients from which susceptibility results were obtained.

In the third-line or later population (*n* = 8), patients with a history of failed first-line national insurance-covered eradication therapy (7 days of triple therapy with VPZ or PPI + AMPC + CAM) and failed second-line national insurance-covered eradication therapy (7 days of triple therapy with VPZ or PPI + AMPC + MNZ) (third-line or later), the eradication rate was 25% (90% CI: 4.6%–60%, *n* = 8) in the ITT analysis and 28.6% (90% CI: 5.3%–65.9%, *n* = 7) in the PP analysis. Among patients with penicillin allergy who did not achieve eradication with AMPC-containing national insurance-covered first- and second-line eradication regimens (*n* = 12), the eradication rate was 100% (90% CI: 77.9%–100%, *n* = 12) in the ITT analysis and 100% (90% CI: 76.2%–100%, *n* = 11) in the PP analysis.

In the third-line or later population (*n* = 8), five patients were available for drug sensitivity testing, and all were MNZ-resistant (100%) and RBT-sensitive. Among patients with penicillin allergy (*n* = 12), all had available culture and sensitivity results; 25% (*n* = 3) were MNZ-resistant and all were RBT-sensitive (*n* = 12).

### 3.4. Adverse Events

The overall incidence of adverse events was 80%, with any adverse event occurring in 16 of the 20 patients. Table 2 lists AEQ scores. Physician assessments did not affect these scores, which were based on each patient’s sensitivities to symptoms. All adverse events including AEQ 2 and 3 were evaluated by physicians, with the vast majority classified as CTCAE grade 1 adverse events. However, two exceptions were identified as the following: one case of CTCAE grade 2 hives and another case of CTCAE grade 2 liver dysfunction. These two CTCAE grade 2 adverse events were not serious and resolved rapidly. The adverse events appeared to be more frequent than those observed than in our previous study using the same AEQ for VPZ, AMPC, and CAM, which are most commonly used in Japan, as well as for VPN, AMPC, and MNZ [18]. All adverse events resolved naturally, and no patients were admitted to the hospital.

## 4. Discussion

This study was the first attempt to evaluate the effectiveness and safety of a triple therapy consisting of RBT 150 mg twice daily, MNZ 250 mg twice daily, and VPZ 20 mg twice daily as a treatment for *H. pylori* rescue eradication [9]. No other interventional studies of *H. pylori* eradication with RBT, MNZ, and VPZ have been reported. We did not achieve the lower limit of the 90% CI exceeding 75% in both the FAS and PPS populations, and the total ITT eradication rate was 70%. In the preplanned subgroup analysis, a 100% eradication rate was observed for MNZ-susceptible *H. pylori*, whereas an insufficient eradication rate of 62.5% was observed for MNZ-resistant *H. pylori*.

In 2021, Gisbert [9] reviewed RBT-containing *H. pylori* eradication regimens, and the mean *H. pylori* eradication rate was 73% (*n* = 3052) in the ITT analysis. Although simple comparisons were difficult to perform because our studies differed in terms of eradication histories and backgrounds of *H. pylori* drug resistance, the ITT analysis of 70% in our study was similar to the overall average reported for RBT-containing regimens. The most commonly reported regimens to date are combinations of RBT, AMPC, and PPIs [24]; others include combinations of RBT, quinolone antibiotics, and PPIs; combinations of RBT, AMPC, bismuth, and PPIs; and combinations of RBT, AMPC, and VPZ [9,16]. In the present study, we established a combination treatment consisting of VPZ, MNZ, and RBT, which had not been previously reported as an intervention study. Therefore, this study is the first to report the eradication success rate of VPZ-MNZ-RBT therapy in cases of sensitivity and resistance to each drug. The absence of RBT-resistant *H. pylori* was an expected finding considering the previously reported resistance rate of only 0.13% (*n* = 9721). A 100% eradication rate was confirmed for MNZ- and RBT-sensitive cases, and these drugs may be promising options for salvage treatment in MNZ- and RBT-sensitive cases. However, the eradication rate for MNZ-resistant and RBT-sensitive cases was 62.5%, which is insufficient and indicates that the presence or absence of MNZ resistance in *H. pylori* is important for predicting the efficacy of VPZ-MNZ-RBT therapy. A systematic review and meta-analysis revealed that tailored therapy based on *H. pylori* drug susceptibility information is effective in eradicating *H. pylori* [25], and VPZ-MNZ-RBT therapy may be an option for tailored therapy for MNZ-susceptible *H. pylori*.

Our study also confirmed resistance information for antibiotics used to eradicate *H. pylori* other than MNZ and RBT. To date, many clinical studies in Japan have reported on the resistance rates of AMPC and CAM [26], but few reports have focused on MNZ, STFX, and RBT [27,28]. We found that AMPC resistance was 0.0% and CAM resistance was 87.5%; AMPC resistance remained low, while CAM resistance was high compared with reported resistance rates before primary eradication. The reasons for low AMPC resistance included the fact that some patients were allergic to penicillin and could not use penicillin, including AMPC, as well as the fact that AMPC resistance was unlikely to develop even if patients had a history of AMPC use. However, the reason for the higher CAM resistance rate in the present study than in previous research (approximately 33%) [5] is due to our recruitment of patients who had previously failed primary eradication. Additionally, our study population included penicillin-allergic patients initially treated with VPZ, CAM, and MNZ [8], many of whom had a history of unsuccessful eradication with CAM. We expected the MNZ resistance rate to be low, but observed a high resistance rate of 50%: this includes patients with failed eradication using MNZ in secondary treatment and those with unsuccessful eradication using VPZ, CAM, and MNZ due to penicillin allergy. We found that the resistance rate to STFX was also high at 68.8%, indicating the need for key antibiotics other than STFX in salvage eradication therapy. Moreover, 37.5% of patients had simultaneous resistance to MNZ, CAM, and STFX, suggesting the need for rescue eradication treatment options other than these three drugs. In such cases, the combination of AMPC and RBT is the most promising option. However, AMPC cannot be used in patients with penicillin allergy, so treatment with a combination of RBT and other antibiotics to which the patient is susceptible is recommended based on culture susceptibility results.

Our study population included eight patients receiving third-line or later therapy and twelve patients with penicillin allergy. The eradication rate was low in the third-line or later group (25% in ITT analysis) and high in the penicillin allergy group (100% in ITT analysis). The main reason for the difference between the groups is likely the difference in their MNZ resistance backgrounds. A previous systematic review and meta-analysis revealed that in cases of MNZ resistance, the eradication rate decreases when eradication is performed with MNZ-containing regimens [29]. We assume the patients in that study had experienced two or more unsuccessful eradication attempts due to factors other than MNZ resistance. The results of this study also suggest that RBT alone may not achieve a sufficient eradication rate for the third-line or later population, even for *H. pylori*, which is susceptible to RBT.

All adverse events observed during our study were classified as either CTCAE grade 1 or, in two cases, CTCAE grade 2. These events were not serious and resolved rapidly, indicating no significant safety concerns. A systematic review of an RBT-containing regimen for *H. pylori* eradication also reported a very low rate of severe adverse events related to RBT [30].

Our study had two main limitations. First, it had a single-center, single-arm interventional design with a small study population. Second, culture susceptibility test results were not available for some patients. Therefore, our findings need to be confirmed in a larger-scale, multicenter, interventional study with culture susceptibility test results. Specifically, we found that in the third-line or later population, MNZ resistance rate was high at 100%, resulting in a low eradication rate (25% in ITT analysis), while in the penicillin allergy population, MNZ resistance rate was low at 25%, resulting in a high eradication rate (100% in ITT analysis).

## 5. Conclusions

We demonstrated a 70% eradication rate for RBT-MNZ-VPZ therapy in ITT analysis as a rescue therapy for *H. pylori* in patients who failed first- and second-line national insurance-covered eradication treatments or those unable to receive these treatments due to penicillin allergy in Japan. The eradication rate was 100% in MNZ- and RBT-susceptible cases and 62.5% in MNZ-resistant but RBT-susceptible cases. These findings suggest the potential of RBT-MNZ-VPZ therapy as a rescue regimen, especially in MNZ- and RBT-susceptible cases, and as a candidate for RBT-containing therapy when AMPC cannot be used because of penicillin allergy.

To further validate the findings of this study, a larger-scale, multicenter, interventional trial is needed to assess the efficacy of RBT-MNZ-VPZ therapy as rescue regimen, incorporating *H. pylori* susceptibility for RBT and MNZ.

## Figures and Tables

**Table 1:** Patient characteristics and *Helicobacter pylori* eradication rates.

Characteristic	Total (*n* = 20)
Age, years, mean ± SE	59 ± 12
Male sex, *n* (%)	7/20 (35.0%)
Cigarette smoking, *n* (%)	2/20 (10.0%)
Alcohol drinking, *n* (%)	8/20 (40.0%)
Height, cm, mean ± SE	161.9 ± 9.5
Weight, kg, mean ± SE	61.9 ± 16.9
BMI, kg/m^2^, mean ± SE	23.4 ± 4.7
T-Bil, mg/dL, mean ± SE	0.75 ± 0.19
AST, U/L, mean ± SE	23.5 ± 8.4
ALT, U/L, mean ± SE	21.5 ± 16.6
Cr, mg/dL, mean ± SE	0.77 ± 0.17
Evaluation by urea breath test, *n* (%)	20/20 (100%)
Endoscopic finding	
Gastritis only, *n* (%)	20/20 (100%)
Diagnosis of *H. pylori* infection ^a^	
UBT, *n* (%)	16/20 (80.0%)
*H. pylori* stool antigen test ^b^, *n* (%)	3/20 (15.0%)
*H. pylori* culture, *n* (%)	1/20 (5.0%)
Drug resistance information available from culture, *n* (%)	16/20 (80.0%)
Resistance to AMPC, *n* (%)	0/16 (0.0%)
AMPC MIC, μg/mL, mean ± SD	0.09 ± 0.08
Resistance to CAM, *n* (%)	14/16 (87.5%)
CAM MIC, μg/mL, mean ± SD	16 ± 9.7
Resistance to MNZ, *n* (%)	8/16 (50.0%)
MNZ MIC, μg/mL, mean ± SD	17.8 ± 21.4
Resistance to STFX, *n* (%)	11/16 (68.8%)
STFX MIC, μg/mL, mean ± SD	0.37 ± 0.65
RBT resistance information available from culture, *n* (%)	15/20 (75.0%)
Resistance to RBT, *n* (%)	0/15 (0.0%)
RBT MIC, μg/mL	All cases < 0.03
Multi-resistance, *n* (%)	
MNZ-R, CAM-R, STFX-R	6/16 (37.5%)
MNZ-R, CAM-R, STFX-S	2/16 (12.5%)
MNZ-R, CAM-S, STFX-R	0/16 (0.0%)
MNZ-R, CAM-S, STFX-S	0/16 (0.0%)
MNZ-S, CAM-R, STFX-R	5/16 (31.3%)
MNZ-S, CAM-R, STFX-S	1/16 (6.3%)
MNZ-S, CAM-S, STFX-R	0/16 (0.0%)
MNZ-S, CAM-S, STFX-S	2/16 (12.5%)
Eradication rate, % (90% CI) (FAS)	70.0% (49.2%–86.0%), *n* = 20
Eradication rate, % (90% CI) (PPS)	72.2% (50.2%–88.4%), *n* = 18

^a^ Diagnosis method for *H. pylori* infection before eradication therapy. ^b^ Eradication success rate determined by the *H. pylori* stool antigen test.

**Table 2:** Adverse effects of treatment with rifabutin, metronidazole, and VPZ triple therapy assessed by AEQ.

	AEQ 1, 2, or 3	AEQ 2 or 3	AEQ 3
Diarrhea	25%	5%	0%
Dysgeusia	15%	5%	0%
Nausea	30%	15%	10%
Anorexia	30%	10%	10%
Abdominal pain	30%	15%	5%
Heartburn	30%	10%	5%
Hives	15%	5%	0%
Headache	30%	15%	10%
Abdominal fullness	40%	5%	0%
Belching	40%	15%	15%
Vomiting	5%	0%	0%
General malaise	5%	0%	0%
Other	25%	10%	10%

AEQ, adverse effects questionnaire; AEQ 1, weak; AEQ 2, moderate; AEQ 3, strong.

## Data Availability

The data that support the findings of this study are available from the corresponding author upon reasonable request.
